# Unusual inheritance of a functional *cki* homolog in the human pathogen *Schistosoma mansoni*

**DOI:** 10.1126/sciadv.aea4905

**Published:** 2025-12-10

**Authors:** George R. Wendt, James J. Collins

**Affiliations:** ^1^ Department of Pharmacology, University of Texas Southwestern Medical Center, Dallas, TX, USA.; ^2^ Howard Hughes Medical Institute, Chevy Chase, MD, USA.

## Abstract

Schistosomes, parasitic flatworms responsible for the neglected tropical disease schistosomiasis, are protected by a skin-like tegument, and tegument maintenance is controlled by a schistosome ortholog (*p53-1*) of the tumor suppressor TP53. To understand *p53-1* function, we characterized a schistosome cyclin-dependent kinase inhibitor homolog (*cki*). Knockdown of *cki* resulted in hyperproliferation that, combined with *p53-1* knockdown, yielded tumor-like growths, indicating that *cki* and *p53-1* are tumor suppressors in *Schistosoma mansoni*. *cki* homologs are ubiquitous in parasitic flatworms but are absent from their free-living ancestors, suggesting that *cki* may have come from horizontal gene transfer. This suggests that the evolution of parasitism in flatworms was aided by an unusual means of metazoan genetic inheritance.

## Teaser

Horizontal gene transfer may have played an important role in the evolution of parasitic flatworms.

## INTRODUCTION

Parasitic flatworms are a diverse clade of organisms that include pathogens of great medical and agricultural purposes. All parasitic flatworms come from a single speciation event and, therefore, are a derived clade relative to all free-living flatworms (*1*). As such, common traits shared across parasitic flatworms could represent early adaptations that facilitated a free-living ancestor in their transition to parasitic lifestyle. One such common trait is the presence of the tegument, a syncytial skin-like tissue that is essential for successful parasitism (*2*). Recent studies in the human pathogen *Schistosoma mansoni* have begun to elucidate the cellular and molecular mechanisms that govern tegument development (*3*, *4*), including evidence that the parasite ortholog of *TP53* (*Sman-p53-1*, *p53-1* for brevity, gene symbol Smp_139530) is a master regulator of tegument development (*5*). TP53 homologs have previously been shown to regulate stem cells and epidermis production in free-living planarians (*6*), in part, via its transcriptional regulation of genes essential for normal epidermis development (*7*). It is unclear how *p53-1* regulates tegument production, but one of the most well-characterized mechanisms of function of TP53 homologs across a variety of animals involves its regulation of the expression of the cyclin-dependent kinase inhibitors (CKIs), proteins that interact with CDK/cyclin complexes to cause cell cycle exit (*8*–*10*). Direct regulation of CKIs by TP53 homologs is well established in vertebrates (*9*, *11*), but the relationship between TP53 homologs and CKIs in invertebrates is less clear (*12*, *13*).

## RESULTS

**Fig. 1.**
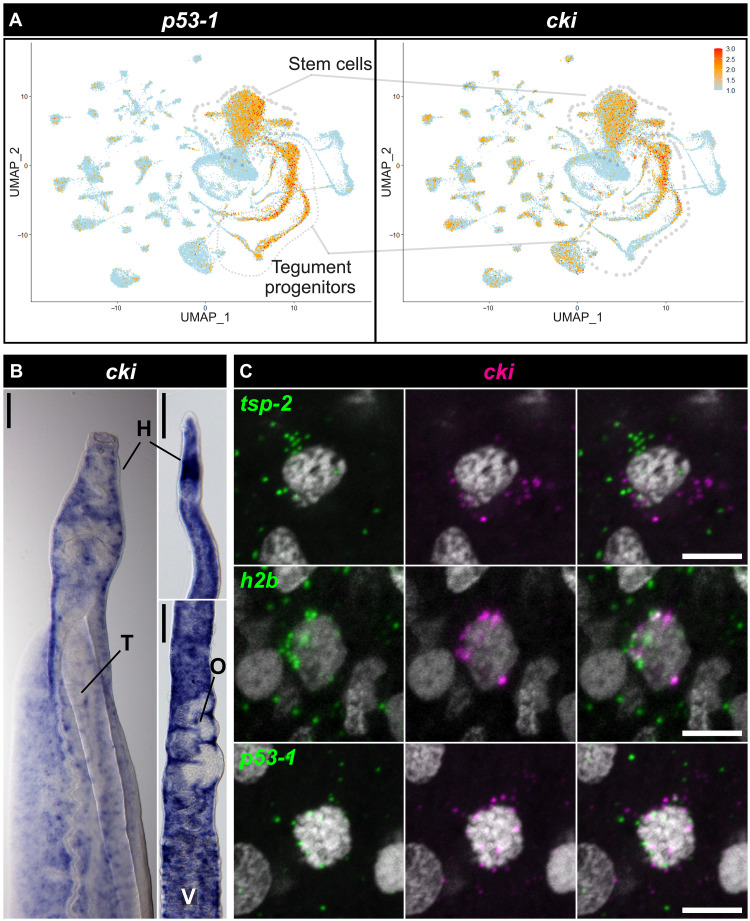
*cki* is coexpressed with the schistosome homolog of *TP53*. (**A**) Uniform manifold approximation plots (UMAP) showing expression patterns of *p53-1* and *cki* in adult schistosomes. Red indicates high expression, orange indicates medium expression, and blue indicates low/no expression. (**B**) Colorimetric whole-mount in situ hybridization (WISH) showing expression patterns of *cki* in male (left) and female (right) worms. H, head; T, testes; O, ovary; V, vitellaria. (**C**) Double fluorescence in situ hybridization (FISH) experiment showing expression of *cki* relative to the tegument progenitor marker *tsp-2*, the proliferative cell marker *h2b*, and *p53-1*. Scale bars, (B) 500 and (C) 5 μm.

Having previously found that *p53-1* (Smp_139530), the schistosome homolog of TP53, plays an important role in tegument development (*5*), we sought to identify the targets of this transcription factor to understand how *p53-1* controls tegument development. We reasoned that *p53-1* and its transcriptional targets would likely have a similar expression pattern. Using our previously generated single-cell RNA-sequencing atlas (*14*), we identified a gene (*Smp_199050*) that was expressed in many of the same cells as *p53-1*, including tegument progenitor cells and stem cells, suggesting that it could play a role in regulating tegument production (Fig. 1, A to C). Although this homolog did not have any close homology to any genes outside of parasitic flatworms, some of its homologs in other parasitic flatworms (e.g., *Clonorchis sinensis* CSKR_104219-T1) were annotated with a cyclin-dependent kinase inhibitor (CDI) superfamily domain [InterPro entry IPR044898; (*15*)]. Upon closer examination, Smp_199050 did bear relatively high similarity and identity to other CDI domain–containing proteins such as *Drosophila* Dap and human CDKN1B (fig. S1, A and B). Additionally, the AlphaFold (*16*) predicted structure of the N terminus of Smp_199050 conformed to the structure of CDKN1B when superimposed on the solved structure of CDK4/CCND1/CDKN1B (fig. S1C) (*17*). As such, we opted to refer to Smp_199050 as *cki* going forward.

**Fig. 2.**
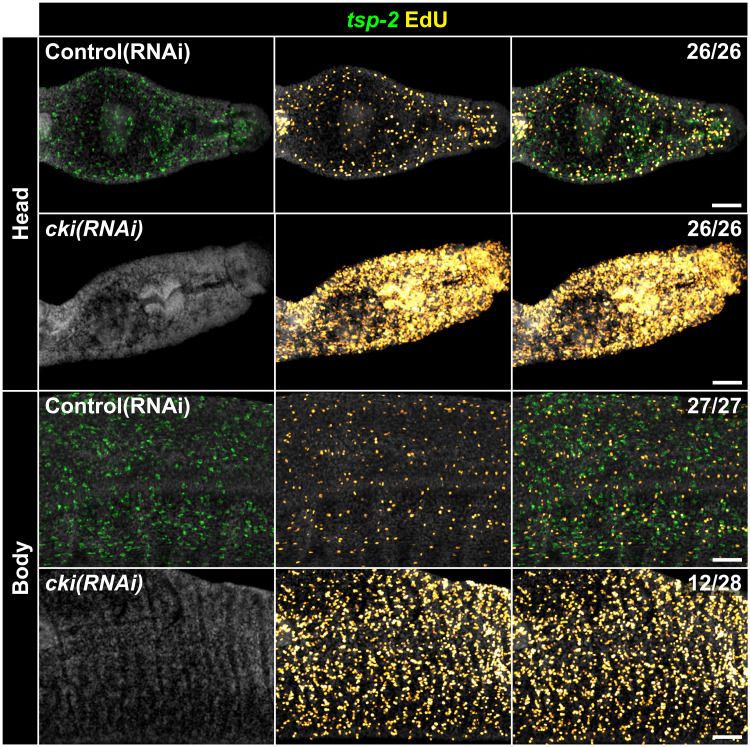
*cki* RNAi results in an increase in proliferation at the expense of differentiation. Representative images of FlSH experiment in conjunction with EdU detection showing expression pattern of the tegument progenitor marker *tsp-2* (green) together with the presence of proliferative EdU^+^ cells (yellow) under the indicated RNAi conditions. The fraction indicates the number of worms that are similar to the representative image. Data are from >26 parasites per treatment from three biological replicates. Scale bar, 50 μm.

We next tested the function of *cki* in the parasite. In other organisms, *cki* homologs act as tumor suppressors, in part, by binding to and inhibiting the cyclin/CDK complexes that control the cell cycle, halting proliferation (*18*). As such, loss of function of *cki* homologs results in hyperproliferation in a variety of animals in a variety of contexts (*19*–*24*). When we knockdown *cki* using RNA interference (RNAi), we similarly observe a hyperproliferation phenotype (Fig. 2 and fig. S2, A and B). While there is nearly 100% penetrance of the hyperproliferation phenotype in the parasite’s head (Fig. 2, top), there is variable penetrance of the phenotype in the body of the worm (Fig. 2, bottom). This increase in proliferation appears to be accompanied by a loss of production of *tsp-2*^+^ tegument progenitor cells (Fig. 2 and fig. S2, A and B) as well as new tegument cells (fig. S2, C and D), demonstrating that the increase in proliferation is accompanied by a loss of differentiation. Additionally, we see an approximately fivefold increase in apoptotic cells in the heads of *cki(RNAi)* parasites (fig. S2, E and F). Together, this suggests that *cki* RNAi causes stem cells to engage in hyperproliferation at the expense of differentiation, ultimately resulting in stem cell death.

**Fig. 3.**
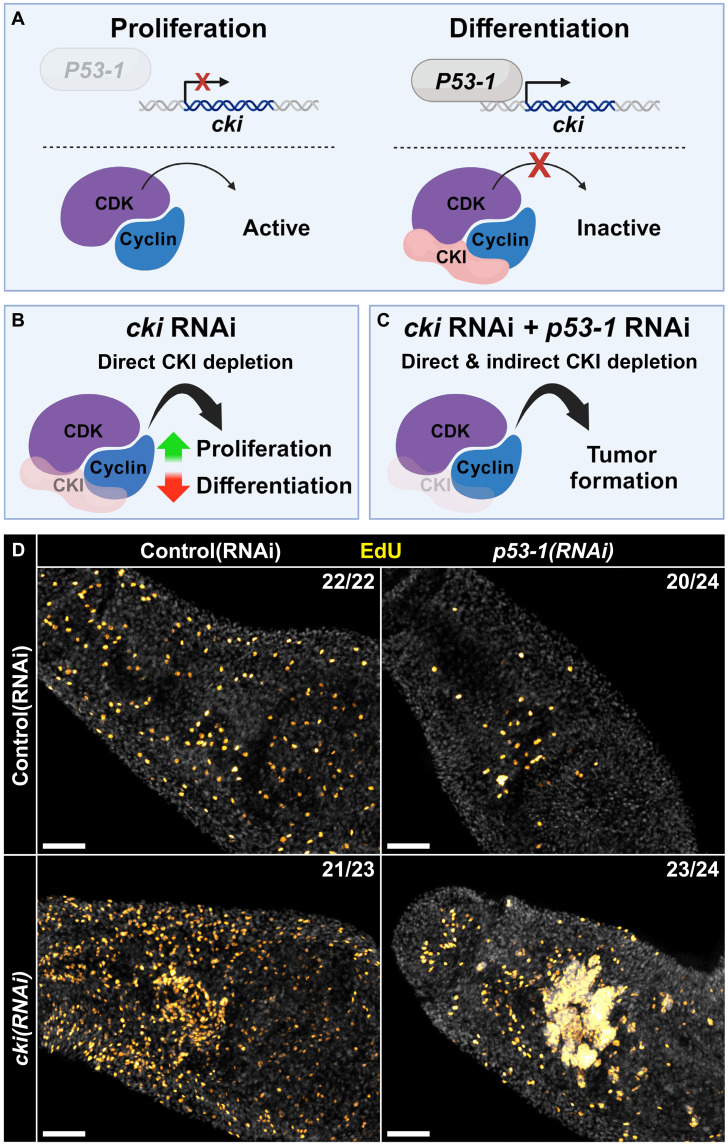
*cki* and *p53-1* cooperate as tumor suppressors in *S. mansoni*. (**A**) Cartoon depicting model of *p53-1* and *cki* interaction in normal parasites. In proliferative cells, P53-1 activity is low and *cki* is not transcribed, resulting in active cyclin/CDK complexes, promoting proliferation. When proliferative cells commit to differentiating, P53-1 activity is high and *cki* is transcribed, resulting in inactive cyclin/CDK/CKI complexes, promoting differentiation. (**B**) Cartoon depicting consequences of *cki* RNAi. By knocking down *cki*, cyclin/CDK complexes are inappropriately active, leading to increased proliferation and decreased differentiation. (**C**) Cartoon depicting model of knocking down *cki* and *p53-1* simultaneously. Knocking down *cki* directly depletes CKI protein. Knocking down *p53-1* may decrease P53-1–dependent induction of *cki* expression. This combination may lower CKI levels low enough that proliferating cells become abnormal and form tumors. (**D**) Representative image of EdU-labeled proliferative cells in parasites treated with control RNAi, *p53-1* RNAi, *cki* RNAi, or a combination of *p53-1* and *cki* RNAi. Data are from >22 parasites per treatment from three biological replicates. The fraction indicates the number of worms that are similar to the representative image. Scale bars, 50 μm.

Human *cki* homologs are widely recognized as tumor suppressors (*18*, *25*), but no studies of invertebrate *cki* homologs have demonstrated spontaneous tumor formation in *cki* loss-of-function models (*20*–*23*). Unlike most invertebrate models studied to date, schistosomes have life spans measured in decades (*26*) and rely upon proliferative somatic cells throughout these long lives (*27*). As such, we wondered whether *cki* could function as a tumor suppressor in schistosomes. Knockdown of *cki* alone causes hyperproliferation (Fig. 2) but never results in robust tumorigenesis. Given our original model that *cki* expression might be induced by *P53-1* (Fig. 3A) and that *cki* RNAi leads to hyperproliferation (Fig. 3B), we next wondered whether knocking down *p53-1* in combination with *cki* would create a more permissive environment for tumor formation in the parasite (Fig. 3C). We found that knocking down *p53-1* in conjunction with *cki* does result in the appearance of abnormal proliferative tumor-like masses in the parasite’s head with nearly 100% penetrance (Fig. 3D). This suggests that *cki* and *p53-1* cooperate as tumor suppressors in *S. mansoni*.

**Fig. 4.**
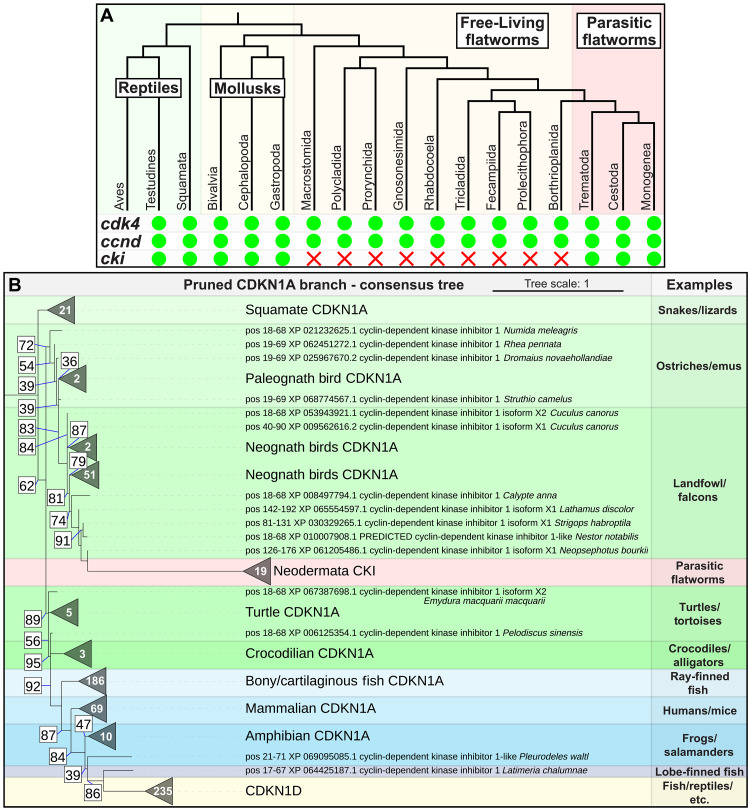
Phylogenetic analysis of parasitic flatworm CKI suggests a horizontal transfer from a distantly related metazoan. (**A**) Top: Cladogram of select metazoans showing the relationship between vertebrates (reptiles) and different invertebrates (mollusks, free-living flatworms, and parasitic flatworms). Bottom: Chart indicating the presence or absence of *cdk4*, *ccnd*, and *cki* homologs in the above species. Green circles indicate presence and red x’s indicate absence. (**B**) Pruned phylogenetic tree (617 members) showing the clade of CDKN1A homologs from the phylogenetic analysis of *S. mansoni* CKI. All parasitic flatworm CKI molecules sit within the avian clade. The white number inside each collapsed branch (triangle) indicates how many members there are in each branch. The number in the white box indicates the UFBoot bootstrap approximation value. Support values for branches with UFBoot support greater than 95 are not shown. Data are from 10 runs.

We were able to readily identify one or more *cki* homologs in nearly all parasitic flatworm genomes (evidence of *cki* homologs present in 57 of the 60 genomes examined) (tables S1 and S2) but were unable to find any *cki* homologs in any free-living flatworm genomes or transcriptomes (present in 0 of the 37 genomes/transcriptomes examined across 10 taxonomic orders) (Fig. 4A and table S2). Additionally, most parasitic flatworm *cki* homologs appear to sit within a microsynteny block downstream of a *TIMM21* homolog and upstream of a *TUBB* homolog (table S1). Examination of annotated free-living flatworm genomes identified no *cki* homologs downstream of their *TIMM21* homologs (table S3), further supporting the absence of *cki* homologs in free-living flatworms. This is unexpected because phylogenetic analysis suggests that all parasitic flatworms arose from a common free-living ancestor several hundred million years ago (*1*). As such, any gene found broadly across parasitic clades should be present at some frequency in the extant free-living flatworms. The fact that we do not observe this suggests either (i) the parasite *cki* homologs arose from de novo gene birth, (ii) *cki* homologs were present broadly throughout all flatworms but were independently lost in all extant free-living flatworm lineages that we have transcriptomic/genomic data for, or (iii) the parasite *cki* homolog was not inherited “vertically” and, therefore, may have originated from horizontal gene transfer (HGT) in an ancestral parasitic flatworm. Given that parasite *cki* homologs have sequence-level similarity to other metazoan *cki* homologs (fig. S1, A and B), de novo gene birth can be safely ruled out. While we cannot rule out multiple independent losses of *cki* homologs/incomplete genomic data across extant free-living flatworms, it would be incredibly unlikely that *cki* homologs were absent from 37 different animals across 10 taxonomic orders, leaving HGT as one of the most likely explanations for the origin of parasitic flatworm *cki* based on phylogenomic analysis.

To further investigate the possibility of HGT, we performed phylogenetic analysis of schistosome *cki* (see Materials and Methods for details). We first obtained a wide-reaching group of CKI homologs related to the schistosome *cki* by performing an iterative DELTA-BLAST search, followed by manual curation of the hits. The vast majority of CKI homologs are largely unstructured except for the CDI domain located in the N terminus [per the InterPro entry for IPR003175; (*15*, *28*)], so we opted to specifically perform the phylogenetic maximum likelihood analysis using the aligned CDI domains. This strategy was largely successful at reconstructing the relationships within vertebrate CKIs (fig. S3): CDKN1A, CDKN1B, CDKN1C, and CDKN1D homologs all clustered with themselves and each other as expected [e.g., CDKN1D is most closely related to CDKN1A (*29*) than to CDKN1B/C] and maintained phylogenetic relationships (Marsupial CDKN1B forms a sister clade to placental mammal CDKN1B). Most invertebrate CKI homologs did not fall neatly within these clades (fig. S3, red asterisks), with most forming a “CKI” clade that does not cluster with any of the well-characterized classes of CKI. Only two exceptions to this exist: First, a small group of spiralians (*Octopus sinensis* and *Helobdella robusta*) cluster together with mammalian CDKN1B (with all other mollusks and annelids falling within the CKI clade), and, second, all parasitic flatworm CKI homologs cluster together with CDKN1A (Fig. 4B; fig. S3, red dashed box; and figs. S4 and S5), specifically within the avian clade, albeit with relatively modest support values. Together with the absence of *cki* homologs in free-living flatworms, these data support HGT as a likely origin for *cki* in parasitic flatworms.

## DISCUSSION

Tumor suppressors such as TP53 and CDKN1A are some of the most widely studied molecules on the planet, but their functions in animals that do not generally develop tumors are largely unexplored. Most glaringly, studies of TP53 homologs in Spiralia have been limited to a handful of species of flatworms. Seminal work in planarians revealed that *Smed-p53* acts as a stem cell regulator and tumor suppressor in planaria (*6*) at least, in part, through regulation of flatworm-specific transcription factors (*7*). Our previous work demonstrated that the schistosome TP53 ortholog, like *Smed-p53*, acts as a stem cell regulator but, unlike *Smed-p53*, does not appear to have any tumor suppressor functions (*5*), leaving open questions as to how TP53 homologs may function in free-living versus parasitic flatworms. Here, we show that schistosomes have a *CDKN1A* homolog, *cki*, that acts as a tumor suppressor together with the parasite’s *TP53* ortholog, *p53-1*. Knocking down this *cki* homolog results in a marked increase in stem cell proliferation with a concomitant decrease in differentiation. Knocking down *cki* in combination with *p53-1* results in spontaneous and robust formation of abnormal proliferative masses of cells, demonstrating that *cki* and *p53-1* are bona fide tumor suppressors in schistosomes.

The exact biology that underlies tumorigenesis upon knockdown of both *p53-1* and *cki* is both fascinating and difficult to study. Knockdown of *cki* alone is sufficient to cause extreme levels of hyperproliferation but does not appear to give rise to tumors. Knockdown of *p53-1*, on the other hand, results in a depletion of proliferative cells and their progeny. Although our model is that knocking down *cki* and *p53-1* at the same time results in both direct (i.e., *cki* RNAi) and indirect (i.e., loss of *p53-1*–mediated *cki* transcription) loss of *cki* transcript, enabling tumorigenesis (Fig. 3, A to C), this model is difficult to directly test. *p53-1* RNAi causes a loss of the stem and progenitor cells (*5*) where *cki* is expressed (Fig. 1A), so we cannot directly test to see if depletion of *p53-1* results in less *cki* transcription. Further studies of *p53-1* transcriptional targets may reveal the exact nature of the interaction between *cki* and *p53-1*.

Our findings also raise questions about the nature of tumor suppressors in flatworms. Why is loss of function of TP53 orthologs sufficient to cause tumorigenesis in free-living flatworms, but tumorigenesis in parasitic flatworms requires a “second hit”? The most obvious difference between these animals is their ecological niche; does the parasitic lifestyle change the fundamental issues that long-lived animals must tackle with respect to tumor suppression? Are there other biological differences between free-living and parasitic flatworms that might be driving these changes [such as the loss of Piwi-interacting RNA (piRNA) in parasitic flatworms; (*30*)]? A comparative examination of the function of “canonical” tumor suppressors in free-living and parasitic flatworms could provide insights into the evolution of tumor suppressors, insights that may have implications outside of the phylum Platyhelminthes. As such, future studies in this direction are of great importance.

Perhaps, one of the most likely origins of the parasite’s *cki* homolog is HGT. Given that parasitic flatworms all derive from a common free-living ancestor (*1*), anything ubiquitous in parasitic flatworms should be present at some frequency in extant free-living flatworms. That *cki* is absent from free-living flatworms led us to three unusual explanations for its origin. First, we believe that we can safely rule out de novo gene birth given that parasite *cki* is related to other metazoan *cki* homologs on the sequence level (fig. S1, A and B). That leaves us with either (i) a situation where *cki* homologs were broadly present throughout all flatworms, but then sometime after parasitic flatworms diverged over 270 million years ago (*31*), the ancestors of the 10 taxonomic orders of free-living flatworms that we have genomic information for lost their *cki* homologs while parasitic flatworms retained theirs; or (ii) a situation where an early ancestral parasitic flatworm acquired a *cki* homolog via HGT. Although maximum likelihood analysis supports an origin for parasitic flatworm *cki* within Aves, we hesitate to definitively assert that parasitic flatworm *cki* derived from any specific clade for a variety of reasons. First, the support values for placement within Aves are relatively modest [UFBoot of 85 upon including nearest-neighbor interchange into phylogenetic analysis (fig. S5)]. Still, parasitic flatworm *cki* homologs consistently fall within CDKN1A homologs (which are only present in vertebrates), supporting a vertebrate origin. Second, our method relies upon alignment of the CDI domain from CKI homologs, which does exclude analysis of the unstructured regions of the gene, which could contain valuable phylogenetic information, but complicate the assumptions that underlie most maximum likelihood analyses (*32*). Third, genomic information for invertebrates (especially spiralians such as flatworms and mollusks) is relatively sparse and low quality compared to the information available for vertebrates. As such, additional genomic information from more spiralian organisms could change our conclusions. We attempted to compensate for this by including additional spiralian *cki* sequences in our maximum likelihood analysis that did not arise from our initial DELTA-BLAST search, but this does not completely eliminate the limitations of spiralian genome sparsity. Last, the donor of parasitic flatworm *cki* would have had to been alive several hundred million years ago (to transmit its *cki* homolog to the ancestral parasitic flatworm) and may not have survived the subsequent mass extinction events to create modern descendants rendering attempts to identify the *cki* donor moot.

Further complicating the assumption that parasite *cki* came from HGT, HGT in metazoans, especially between metazoans, is relatively rare (*33*, *34*). Still, HGT in parasitic flatworms might be more common than in other metazoans. HGT between multicellular organisms has been frequently identified between parasitic plants and their host plants (*35*–*38*). Aside from the analogous relationship between parasitic flatworms and their metazoan hosts, parasitic flatworms may represent especially molecularly fertile ground for HGT. In addition to their syncytial tegument, all parasitic flatworms are also united by their loss of the piRNA pathway (*30*) that, combined with the apparent lack of DNA methylation across flatworms (*39*–*41*), makes the parasitic flatworms deficient in two key pathways used to suppress mobile selfish genetic elements like transposons and endogenous retroviruses (*33*, *42*, *43*). This type of HGT has been reported between parasitic lamprey and their host (*44*), both of which have intact defenses against selfish genetic elements. Selfish genetic element–mediated HGT could, therefore, be unusually successful in parasitic flatworms.

Origin of the *cki* gene notwithstanding, the fact that it is nearly ubiquitous across all parasitic flatworms means that it has been present in parasitic flatworms from early in their evolution and has likely been retained because it plays an important role in these animals. Understanding that role could teach us not only about the evolution of parasitism in these fascinating animals but could also reveal ways to combat the diseases that they cause.

## MATERIALS AND METHODS

### Parasite acquisition and culture

Adult *S. mansoni* (NMRI strain, 6 to 7 weeks postinfection) were obtained from infected female mice by hepatic portal vein perfusion with 37°C Dulbecco’s modified Eagle’s medium (Sigma-Aldrich, St. Louis, MO) plus 10% serum (either fetal calf serum or horse serum) and heparin. Parasites were cultured as previously described (*3*). Unless otherwise noted, all experiments were performed with male parasites to maximize the amount of somatic tissue present and to avoid additional experimental modifications that have to be undertaken for successful in vitro female parasite culture (*45*). Experiments with and care of vertebrate animals were performed in accordance with protocols approved by the Institutional Animal Care and Use Committee of UT Southwestern Medical Center (approval APN: 2017-102092).

### Labeling and imaging

Schistosome colorimetric and fluorescence in situ hybridization analyses were performed as previously described (*3*–*5*, *27*) with the following modification. To improve signal to noise for colorimetric and fluorescence in situ hybridization, worms were incubated at 95°C for 15 min in 10 mM sodium citrate (pH 6.0) before proteinase K digestion. In vitro EdU (5-ethynyl-2′-deoxyuridine, Cayman Chemical 20518) labeling and detection were performed as previously described (*27*). Terminal deoxynucleotidyl transferase–mediated deoxyuridine triphosphate nick end labeling (TUNEL) labeling was carried out as follows: Formaldehyde-fixed worms stored in 100% methanol (MeOH) were rehydrated in 50% MeOH/50% phosphate-buffered saline (PBS) and 0.3% Triton X-100 (pH 7.4; PBSTx) and rinsed in PBSTx. Parasites were then photobleached as described previously (*3*). After photobleaching, parasites were rinsed in PBSTx and then incubated at 95°C for 15 min in 10 mM sodium citrate (pH 6.0). Following this, parasites were rinsed in PBSTx and then permeablized in proteinase K (5 μg/ml; Ambion AM2546). Following proteinase K treatment, parasites were postfixed in 4% formaldehyde in PBSTx. Parasites were then rinsed in PBSTx and then incubated at 37°C in terminal deoxynucleotidyl transferase (TdT) reaction buffer from a Click-iT Plus TUNEL Assay for In Situ Apoptosis Detection kit (Thermo Fisher Scientific, C10617) for 1 hour. Parasites were then incubated at 37°C in TdT reaction mix for 1 hour per the manufacturer’s instructions. Parasites were then thoroughly rinsed, and EdU detection was carried out as previously described (*3*). All fluorescently labeled parasites were counterstained with 4′,6-diamidino-2-phenylindole (1 μg/ml), cleared in 80% glycerol, and mounted on slides with Vectashield (Vector Laboratories).

Confocal imaging of fluorescently labeled samples was performed on a Zeiss LSM900 Laser Scanning Confocal Microscope. Unless otherwise mentioned, all fluorescence images represent maximum intensity projection plots. To perform cell counts, cells were manually counted in maximum intensity projection plots derived from confocal stacks. Bright-field images were acquired on a Zeiss AxioZoom V16 equipped with a transmitted light base and a Zeiss AxioCam 105 color camera.

### RNA interference

All RNAi experiments used freshly perfused male parasites between 6 and 7 weeks postinfection (separated from females). Double-stranded RNA (dsRNA) treatments were all carried out at 30 μg/ml in Basch Media 169. Double-knockdown experiments were carried out at 60 μg/ml (30 μg/ml of each dsRNA) for the first 4 days and then switched to 30 μg/ml (15 μg/ml of each dsRNA) for the remainder of the experiment. dsRNA was generated by in vitro transcription and was replaced daily for 3 days and then every other day until the end of the experiment (14 days for all experiments except for EdU pulse-chase experiments, which were EdU pulsed at day 14 and then carried out for 8 more days). EdU pulses were performed at 5 μM for 4 hours before either fixation or chase as described above.

As a negative control for RNAi experiments, we used a nonspecific dsRNA containing two bacterial genes (*46*). cDNAs used for RNAi and in situ hybridization analyses were cloned as previously described (*46*); oligonucleotide primer sequences are listed in table S4.

### Phylogenetic analysis

Clustal alignment of CKI sequences shown in fig. S1A was performed using Clustal Omega Multiple Sequence Alignment (*47*) (default settings except ORDER was set to “input”) with sequences corresponding to residues 14 to 48 of Smp_199050. Protein sequences used for alignment and species abbreviations can be found in table S5.

Domain structures of CKI homologs shown in fig. S1B were obtained using SMART v9.0 (http://smart.embl-heidelberg.de/) (*48*, *49*).

Percent identity/similarity was determined using EMBOSS Needle PSA (*47*, *50*) (default settings except matrix was set to BLOSUM45) by querying Smp_199050 (*S. mansoni* CKI) versus ECG_07297 (*Echinococcus granulosus* CKI), CG1772 (*Drosophila melanogaster* Dap), or CDKN1B (*Homo sapiens* CDKN1B).

Generation of the superimposition of schistosome *cki* on the solved structure of human CDK4/CCND/CDKN1B shown in fig. S1C was carried out using ChimeraX (*51*) (v1.7.1 2024-01-22) with the Protein Data Bank entry 6p8e (*17*) and residues 1 to 70 of the AlphaFold (*16*, *52*) prediction of Smp_199050 (AF-A0A3Q0KUT0-F1-v4) using Matchmaker with default settings.

Identification of homologs of *S. mansoni cki* (see table S1) was initially carried out using WormBase ParaSite (https://parasite.wormbase.org/) [version 19.0 (March 2024)] (*53*, *54*). Examination of *cki* (Smp_199050) homologs initially only revealed *cki* homologs in trematodes (present in 27 of the 28 unique genomes) and monogeneans (present in one of the three unique genomes). A reciprocal BLAST search of the *Hymenolepis diminuta* genome identified the *cki* homolog WMSIL1_LOCUS6166 (“CDI domain–containing protein”). WormBase ParaSite then identified 12 additional orthologs of WMSIL1_LOCUS6166 in 12 unique cestode genomes, each of which was confirmed to be a *cki* ortholog via a reciprocal BLAST search. Additional BLAST searches of cestode genomes revealed four additional *cki* homologs (or evidence of unannotated homologs) in cestodes (in total present in 16 of the 16 unique genomes), with two being incorrectly included in the gene model of TIMM21 homologs. Further examination of cestode genomes revealed that a second *cki* paralog was present in all cestode genomes except for the genomes of the order Diphyllobothriidea (*Dibothriocephalus latus*, *Schistocephalus solidus*, and *Spirometra erinaceieuropaei*), so we opted to name Smp_199050 orthologs *cki-1* and cestode-specific paralogs *cki-2*. Next, PlanMine v3.0 (https://planmine.mpinat.mpg.de/planmine/) (*55*) was used to search 19 free-living flatworm transcriptomes for *cki* homologs. Further searches of unannotated flatworm genomes were carried out using NCBI genome datasets (www.ncbi.nlm.nih.gov/datasets/genome/) by searching for taxid 6157. Identification of potential CKI homologs was then carried out using tblastn (default settings except matrix was set to BLOSUM45) with queries of Smp_199050 (*S. mansoni* CKI), PXEA_0000526601 (*Protopolystoma xenopodis* CKI), and HmN_000277000 (*Hymenolepis nana* CKI). The unannotated genomes searched, organism clade, genome accession number, scaffold hit from the tblastn search, and E-value of each hit are listed in table S2.

Curation and alignment of CKI protein sequences were carried out by first performing a DELTA-BLAST (*56*) (version 2.12.0+) search with a query of Sman-CKI against the NCBI “nr_cluster” database (downloaded on 19 November 2024) with the following parameters: -evalue 0.001 -num_threads 4 -num_iterations 5 -matrix BLOSUM45 -max_target_seqs 10000 (see table S6). The hits from the third iteration (shaded green in table S6) were then filtered by removing all non-RefSeq (*57*) sequences (i.e., retaining only sequences with accessions beginning with “XP_” or “NP_”), yielding 1381 hits from 1380 unique accessions. To ensure better representation of spiralian and parasitic flatworm sequences, we also added additional RefSeq spiralian CKI homologs (21 additional homologs, see table S7) and non-RefSeq parasitic flatworm homologs [52 additional homologs that (i) have accessions that do not begin with “XP_,” (ii) have correct or correctable gene models for *cki* (*D. latus* CKI was excluded for this reason), and (iii) have unique accessions (a recent duplication in the *Hymenolepis microstoma* genome results in two different CKI-2 homologs with identical amino acid sequences and the same accession); see table S1], creating a final total of 1453 sequences. We then used the LAST feature (updated 22 December 2022) of MAFFT (*58*, *59*) (version 7) to extract and align the CDI domains from our CKI homologs using the CDI domain of human CDKN1B (residues 29 to 72) as the reference region, with default settings except that “minimum coverage” was set to 0.3. This resulted in 1411 sequences (see table S8), with most attrition happening to parasitic flatworm CKI homologs; 36 of the 42 sequences that were lost were from parasitic flatworms. Even with this attrition, we still retained 19 of the 55 parasitic flatworm CKI homologs, which we reasoned would be enough to perform robust analysis.

Maximum likelihood analysis and phylogenetic tree construction was carried out using IQ-TREE (*60*) (IQ-TREE multicore version 2.4.0 for Linux x86 64-bit built 7 February 2025) with the following settings: -m TEST -bb 1000 -alrt 1000 -T AUTO --runs 10 -nm 2000 and a starting seed of 289843 for Fig. 4 and figs. S3 and S4. The analysis in fig. S5 was carried out using IQ-TREE (*60*) (IQ-TREE multicore version 2.4.0 for Linux x86 64-bit built 7 February 2025) with the following settings: -m TEST -bb 1000 -alrt 1000 -T AUTO --runs 10 -nm 5000 -bnni and a starting seed of 194082. ModelFinder on auto settings selected “Q.plant+I + G4” as the best-fit model. The consensus tree was then uploaded to Interactive Tree of Life (*61*) (https://itol.embl.de/) where it was manually annotated. Bootstrap values are either UFBoot (*62*) values or SH-aLRT (*63*) values as indicated in the figure legends. The unannotated consensus and maximum likelihood trees with and without nearest-neighbor interchange correction in Newick format are available in data S1 to S4.

### Statistical analysis

Statistical analysis was carried out using one-way analysis of variance (ANOVA) tests (quantitative polymerase chain reaction experiments) or two-way ANOVA with Tukey’s multiple comparisons tests (all other experiments). All statistical tests were performed using GraphPad Prism v10.2.3.
