# Supplementary material for: Unusual inheritance of a functional cki homolog in the human pathogen Schistosoma mansoni
Source: Sci Adv. 2025 Dec 10;11(50):eaea4905. doi: 10.1126/sciadv.aea4905 (PMC12693960; doi:10.1126/sciadv.aea4905)
Supplement: Supplementary file 1 — Figs. S1 to S5 Legends for tables S1 to S8 Legends for data S1 to S4 [file sciadv.aea4905_sm.pdf]

Science Advances
AAAS

Supplementary Materials for

Unusual inheritance of a functional *cki* homolog in the human pathogen *Schistosoma mansoni*

George R. Wendt and James J. Collins III

Corresponding author: George R. Wendt, george.wendt@utsouthwestern.edu;
James J. Collins, jamesj.collins@utsouthwestern.edu

Sci. Adv. **11**, eaea4905 (2025)
DOI: 10.1126/sciadv.aea4905

The PDF file includes:

Figs. S1 to S5
Legends for tables S1 to S8
Legends for data S1 to S4

Other Supplementary Material for this manuscript includes the following:

Tables S1 to S8
Data S1 to S4

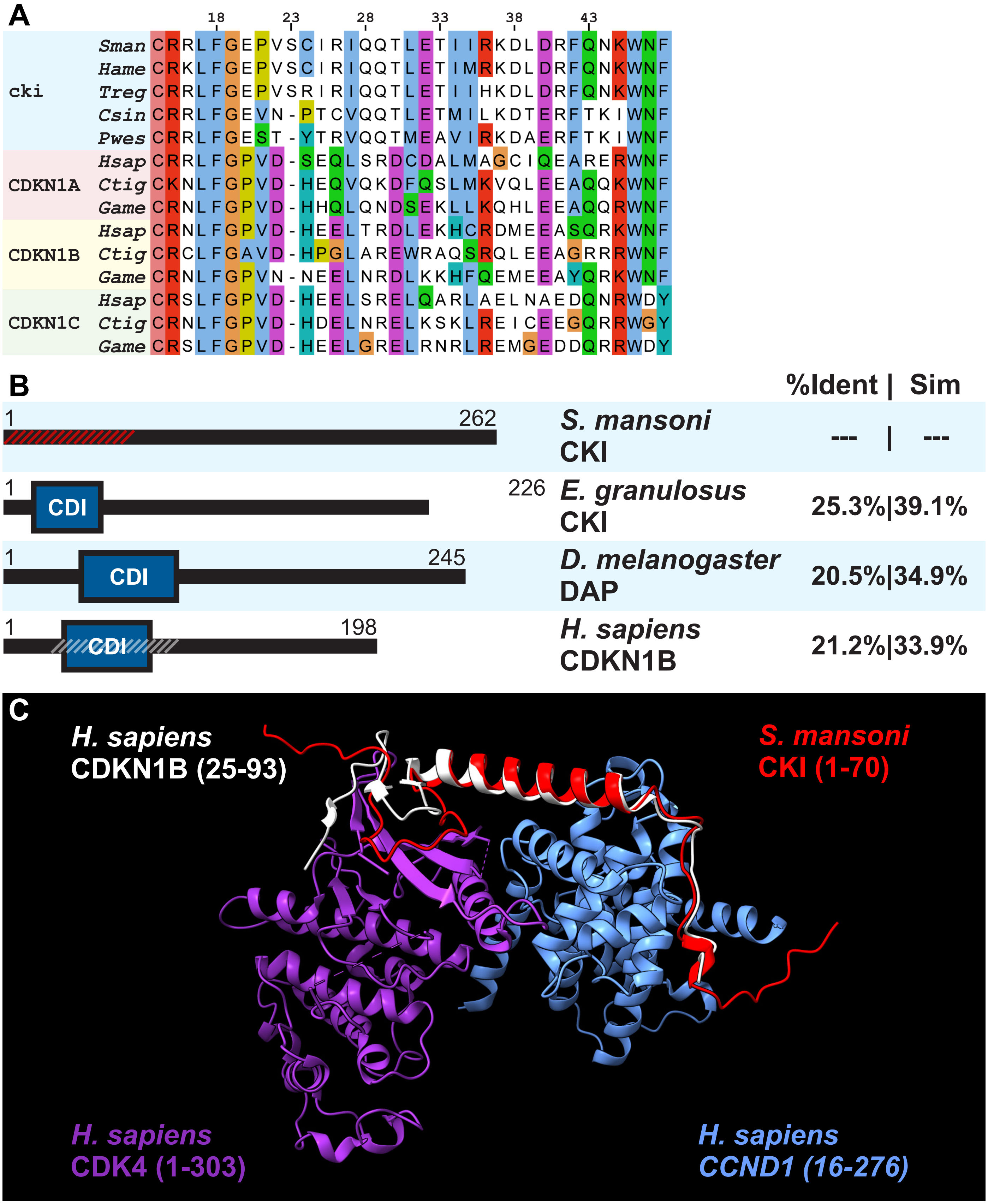
A
18 23 28 33 38 43
cki
Sman CRRLFGEPVSCIRIQQTLETIIRKDLDRFQNKWNF
Hame CRKLFGEPVSCIRIQQTLETIMRKDLDRFQNKWNF
Treg CRRLFGEPVSRIRIQQTLETIIHKDLDRFQNKWNF
Csin CRRLFGEVN-PTCVQQTLETMILKDTERFTKIWNF
Pwes CRRLFGEST-YTRVQQTMEAVIRKDAERFTKIWNF
CDKN1A
Hsap CRRLFGPVD-SEQLSRDCDALMAGCIQEARERWNF
Ctig CKNLFGPVD-HEQVQKDFQSLMKVQLEEAQQKWNF
Game CRNLFGPVD-HHQLQNDSEKLLKQHLEEAQQRWNF
CDKN1B
Hsap CRNLFGPVD-HEELTRDLEKHCRDMEEASQRKWNF
Ctig CRCLFGAVD-HPGLAREWRAQSRQLEEAGRRRWNF
Game CRNLFGPVN-NEELNRDLKKHFQEMEEAYQRKWNF
CDKN1C
Hsap CRSLFGPVD-HEELSRELQARLAELNAEDQNRWDY
Ctig CRNLFGPVD-HDELNRELKSKLREICEEGQRRWGY
Game CRSLFGPVD-HEELGRELRNRLREMGEDDQRRWDY
B
%Ident | Sim
1 262 S. mansoni CKI --- | ---
1 CDI 226 E. granulosus CKI 25.3%|39.1%
1 CDI 245 D. melanogaster DAP 20.5%|34.9%
1 CDI 198 H. sapiens CDKN1B 21.2%|33.9%
C
H. sapiens CDKN1B (25-93)
S. mansoni CKI (1-70)
H. sapiens CDK4 (1-303)
H. sapiens CCND1 (16-276)

Fig. S1.

Figure S1. *Schistosoma mansoni* CKI protein domains/structure. (A) Clustal alignment of *Schistosoma mansoni* (*Sman*) CKI (Smp_199050) residues 14-48 with CKI sequences from a variety of parasitic flatworms (*Heterobilharzia americana – Hame*, *Trichobilharzia regenti – Treg*, *Clonorchis sinensis – Csin*, *Paragonimus westermani – Pwes*) as well as CDKN1A, CDKN1B, and CDKN1C sequences from humans (*Hsap*), tiger rattlesnakes (*Ctig*), and whooping cranes (*Game*). The number at top of alignment corresponds to position in the alignment relative to the *Schistosoma mansoni* CKI. Protein sequences used for alignment can be found in **Table S5** (B) Schematic of the domain structure of CKI homologs from *S. mansoni*, *E. granulosus*, *D. melanogaster*, and *H. sapiens* along with the percentage identity/similarity relative to the *S. mansoni* CKI homolog. (C) Superimposition of the AlphaFold indicated residues of *S. mansoni* CKI (accession: AF_A0A3Q0KUT0-F1-model_v4) onto the solved structure of *H. sapiens* CDKN1B, CCND1, and CDK4 (PDB accession: 6p8e). Hash-shaded portion from (B) corresponds to the superimposition in (C).

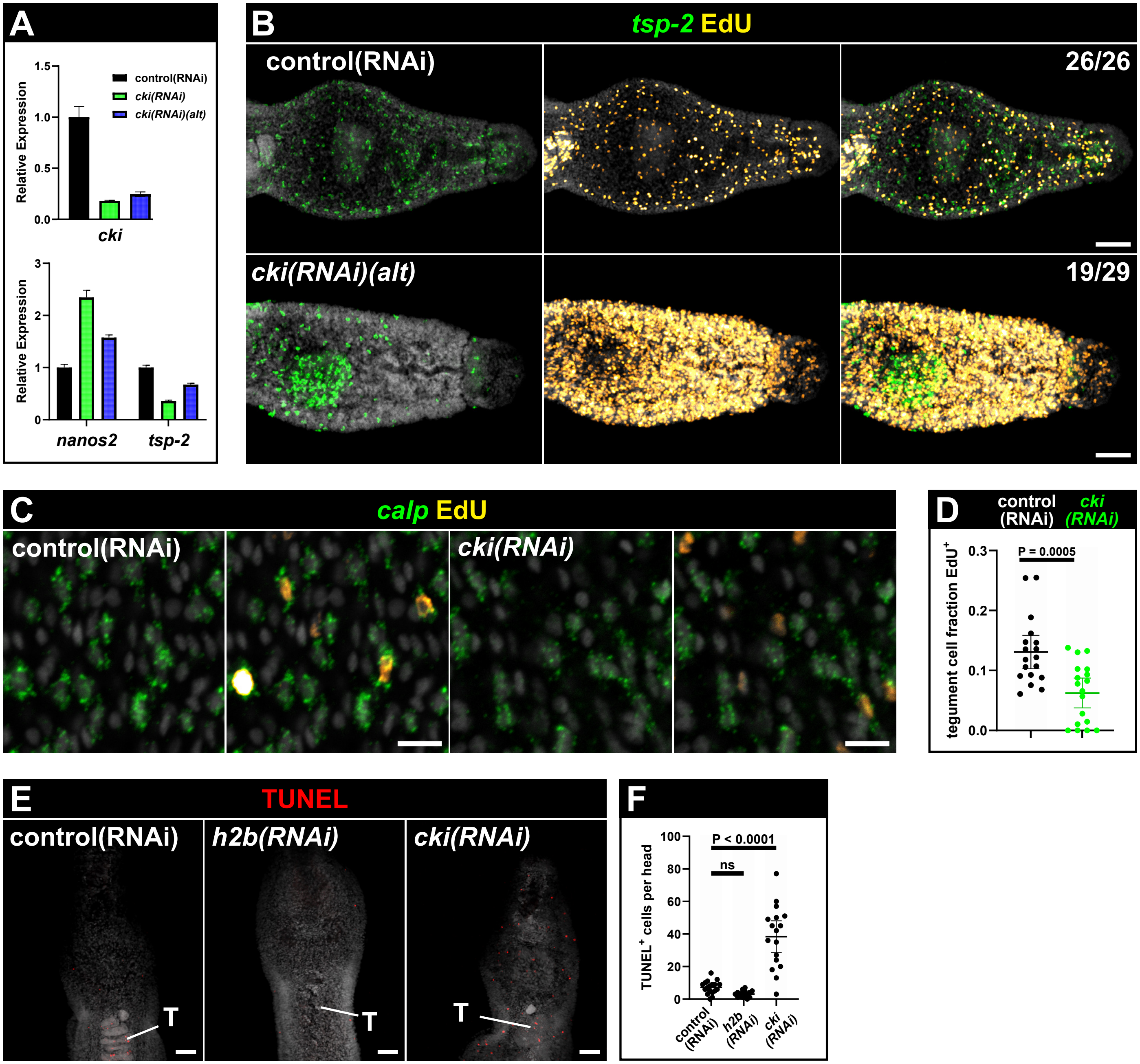
A
Relative Expression
control(RNAi)
cki(RNAi)
cki(RNAi)(alt)
cki
nanos2
tsp-2
B
tsp-2 EdU
control(RNAi)
26/26
cki(RNAi)(alt)
19/29
C
calp EdU
control(RNAi)
cki(RNAi)
D
control (RNAi)
cki (RNAi)
P = 0.0005
tegument cell fraction EdU+
E
TUNEL
control(RNAi)
h2b(RNAi)
cki(RNAi)
T
F
P < 0.0001
ns
TUNEL+ cells per head
control (RNAi)
h2b (RNAi)
cki (RNAi)

Fig. S2.

Figure S2. *cki* RNAi results in an increase in proliferation at the expense of differentiation. (A) qPCR showing expression of *cki*, the stem cell marker *nanos2*, and the tegument progenitor marker *tsp-2* under indicated RNAi conditions. 'cki(RNAi)(alt)' indicates knockdown performed with dsRNA corresponding to a non-overlapping sequence relative to the original dsRNA sequence. Data are from one biological replicate. (B) FlSH experiment in conjunction with EdU detection showing expression pattern of the tegument progenitor marker *tsp-2* (green) in conjunction with the presence of proliferative EdU^+ cells (yellow) under the indicated RNAi conditions. Note that the micrographs for control(RNAi) are duplicates of the micrographs for control(RNAi) from Figure 3 (top) as these came from the same experiment. Data are from >26 parasites/treatment from three biological replicates. (C) EdU pulse-chase experiment combined with FISH of the tegument marker *calp* after control or *cki* RNAi. Neoblast progeny are labeled with EdU after an 8-day chase. (D) Quantification of (C). Data are from 18 parasites/treatment from two biological replicates. (E) Fluorescent TUNEL assay labeling DNA breaks in worms under the indicated RNAi conditions. T, testes. Scale bar: 50 µm. Note signal in the testes is a normal occurrence in the parasite's germline. (F) Quantification of (E). Data are from >16 parasites/treatment from three biological replicates. Scale bars: (B) 50 µm, (C) 10 µm. (E) 50 µm.

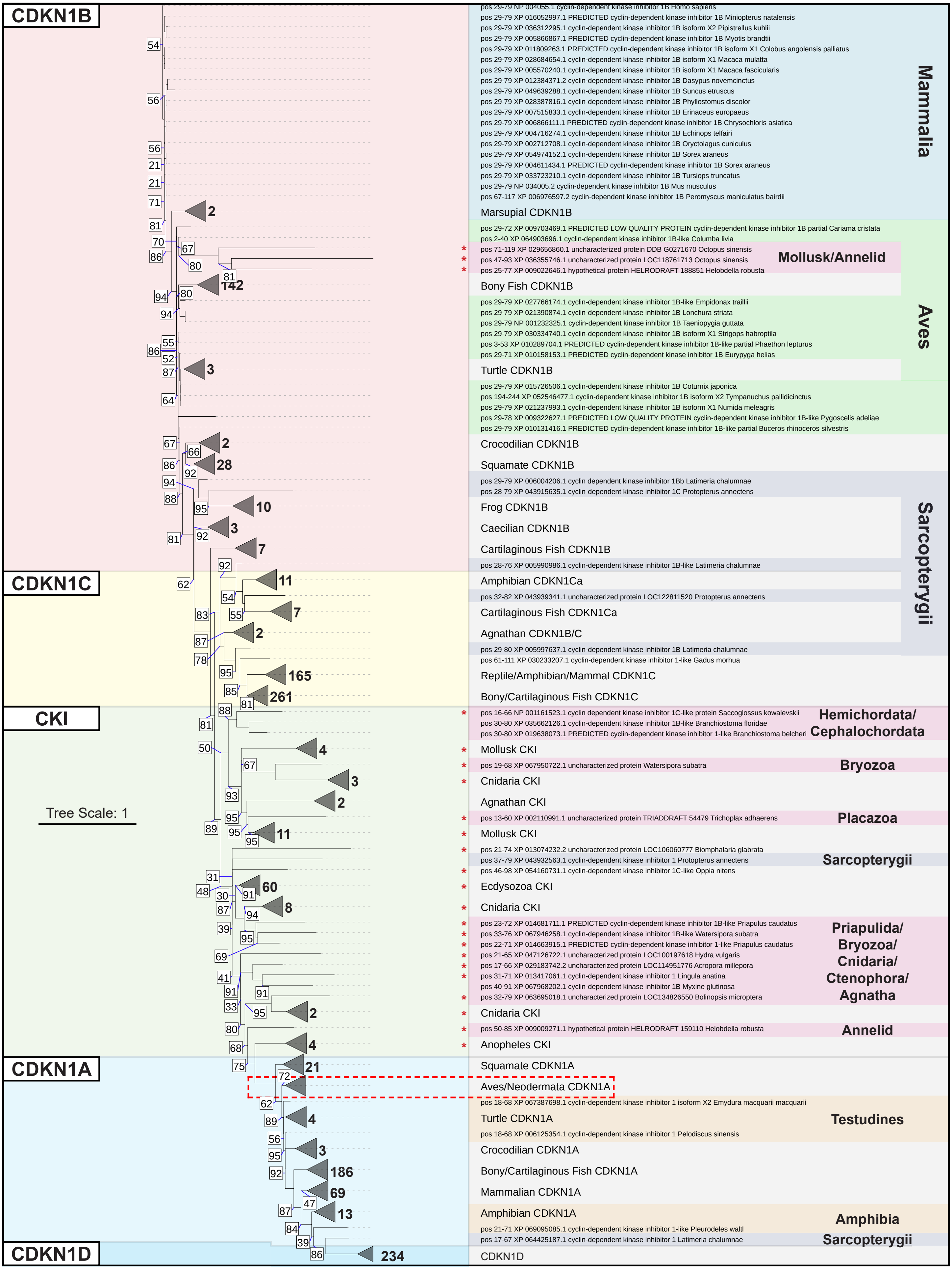
CDKN1B
CDKN1C
CKI
CDKN1A
CDKN1D
Tree Scale: 1
pos 29-79 NP 004055.1 cyclin-dependent kinase inhibitor 1B Homo sapiens
pos 29-79 XP 016052997.1 PREDICTED cyclin-dependent kinase inhibitor 1B Miniopterus natalensis
pos 29-79 XP 036312295.1 cyclin-dependent kinase inhibitor 1B isoform X2 Pipistrellus kuhlii
pos 29-79 XP 005866867.1 PREDICTED cyclin-dependent kinase inhibitor 1B Myotis brandtii
pos 29-79 XP 011809263.1 PREDICTED cyclin-dependent kinase inhibitor 1B isoform X1 Colobus angolensis palliatus
pos 29-79 XP 028684654.1 cyclin-dependent kinase inhibitor 1B isoform X1 Macaca mulatta
pos 29-79 XP 005570240.1 cyclin-dependent kinase inhibitor 1B isoform X1 Macaca fascicularis
pos 29-79 XP 012384371.2 cyclin-dependent kinase inhibitor 1B Dasypus novemcinctus
pos 29-79 XP 049639288.1 cyclin-dependent kinase inhibitor 1B Suncus etruscus
pos 29-79 XP 028387816.1 cyclin-dependent kinase inhibitor 1B Phyllostomus discolor
pos 29-79 XP 007515833.1 cyclin-dependent kinase inhibitor 1B Erinaceus europaeus
pos 29-79 XP 006866111.1 PREDICTED cyclin-dependent kinase inhibitor 1B Chrysochloris asiatica
pos 29-79 XP 004716274.1 cyclin-dependent kinase inhibitor 1B Echinops telfairi
pos 29-79 XP 002712708.1 cyclin-dependent kinase inhibitor 1B Oryctolagus cuniculus
pos 29-79 XP 054974152.1 cyclin-dependent kinase inhibitor 1B Sorex araneus
pos 29-79 XP 004611434.1 PREDICTED cyclin-dependent kinase inhibitor 1B Sorex araneus
pos 29-79 XP 033723210.1 cyclin-dependent kinase inhibitor 1B Tursiops truncatus
pos 29-79 NP 034005.2 cyclin-dependent kinase inhibitor 1B Mus musculus
pos 67-117 XP 006976597.2 cyclin-dependent kinase inhibitor 1B Peromyscus maniculatus bairdii
Marsupial CDKN1B
Mammalia
pos 29-72 XP 009703469.1 PREDICTED LOW QUALITY PROTEIN cyclin-dependent kinase inhibitor 1B partial Cariama cristata
pos 2-40 XP 064903696.1 cyclin-dependent kinase inhibitor 1B-like Columba livia
pos 71-119 XP 029656860.1 uncharacterized protein DDB G0271670 Octopus sinensis
pos 47-93 XP 036355746.1 uncharacterized protein LOC118761713 Octopus sinensis
pos 25-77 XP 009022646.1 hypothetical protein HELRODRAFT 188851 Helobdella robusta
Mollusk/Annelid
Bony Fish CDKN1B
pos 29-79 XP 027766174.1 cyclin-dependent kinase inhibitor 1B-like Empidonax traillii
pos 29-79 XP 021390874.1 cyclin-dependent kinase inhibitor 1B Lonchura striata
pos 29-79 NP 001232325.1 cyclin-dependent kinase inhibitor 1B Taeniopygia guttata
pos 29-79 XP 030334740.1 cyclin-dependent kinase inhibitor 1B isoform X1 Strigops habroptila
pos 3-53 XP 010289704.1 PREDICTED cyclin-dependent kinase inhibitor 1B-like partial Phaethon lepturus
pos 29-71 XP 010158153.1 PREDICTED cyclin-dependent kinase inhibitor 1B Eurypyga helias
Turtle CDKN1B
Aves
pos 29-79 XP 015726506.1 cyclin-dependent kinase inhibitor 1B Coturnix japonica
pos 194-244 XP 052546477.1 cyclin-dependent kinase inhibitor 1B isoform X2 Tympanuchus pallidicinctus
pos 29-79 XP 021237993.1 cyclin-dependent kinase inhibitor 1B isoform X1 Numida meleagris
pos 29-78 XP 009322627.1 PREDICTED LOW QUALITY PROTEIN cyclin-dependent kinase inhibitor 1B-like Pygoscelis adeliae
pos 29-79 XP 010131416.1 PREDICTED cyclin-dependent kinase inhibitor 1B-like partial Buceros rhinoceros silvestris
Crocodilian CDKN1B
Squamate CDKN1B
pos 29-79 XP 006004206.1 cyclin dependent kinase inhibitor 1Bb Latimeria chalumnae
pos 28-79 XP 043915635.1 cyclin-dependent kinase inhibitor 1C Protopterus annectens
Frog CDKN1B
Caecilian CDKN1B
Cartilaginous Fish CDKN1B
pos 28-76 XP 005990986.1 cyclin-dependent kinase inhibitor 1B-like Latimeria chalumnae
Amphibian CDKN1Ca
pos 32-82 XP 043939341.1 uncharacterized protein LOC122811520 Protopterus annectens
Cartilaginous Fish CDKN1Ca
Agnathan CDKN1B/C
pos 29-80 XP 005997637.1 cyclin-dependent kinase inhibitor 1B Latimeria chalumnae
Sarcopterygii
pos 61-111 XP 030233207.1 cyclin-dependent kinase inhibitor 1-like Gadus morhua
Reptile/Amphibian/Mammal CDKN1C
Bony/Cartilaginous Fish CDKN1C
pos 16-66 NP 001161523.1 cyclin dependent kinase inhibitor 1C-like protein Saccoglossus kowalevskii
pos 30-80 XP 035662126.1 cyclin-dependent kinase inhibitor 1B-like Branchiostoma floridae
pos 30-80 XP 019638073.1 PREDICTED cyclin-dependent kinase inhibitor 1-like Branchiostoma belcheri
Hemichordata/ Cephalochordata
Mollusk CKI
pos 19-68 XP 067950722.1 uncharacterized protein Watersipora subatra
Bryozoa
Cnidaria CKI
Agnathan CKI
pos 13-60 XP 002110991.1 uncharacterized protein TRIADDRAFT 54479 Trichoplax adhaerens
Placazoa
Mollusk CKI
pos 21-74 XP 013074232.2 uncharacterized protein LOC106060777 Biomphalaria glabrata
pos 37-79 XP 043932563.1 cyclin-dependent kinase inhibitor 1 Protopterus annectens
Sarcopterygii
pos 46-98 XP 054160731.1 cyclin-dependent kinase inhibitor 1C-like Oppia nitens
Ecdysozoa CKI
Cnidaria CKI
pos 23-72 XP 014681711.1 PREDICTED cyclin-dependent kinase inhibitor 1B-like Priapulus caudatus
pos 33-76 XP 067946258.1 cyclin-dependent kinase inhibitor 1B-like Watersipora subatra
pos 22-71 XP 014663915.1 PREDICTED cyclin-dependent kinase inhibitor 1-like Priapulus caudatus
pos 21-65 XP 047126722.1 uncharacterized protein LOC100197618 Hydra vulgaris
pos 17-66 XP 029183742.2 uncharacterized protein LOC114951776 Acropora millepora
pos 31-71 XP 013417061.1 cyclin-dependent kinase inhibitor 1 Lingula anatina
pos 40-91 XP 067968202.1 cyclin-dependent kinase inhibitor 1B Myxine glutinosa
pos 32-79 XP 063695018.1 uncharacterized protein LOC134826550 Bolinopsis microptera
Priapulida/ Bryozoa/ Cnidaria/ Ctenophora/ Agnatha
Cnidaria CKI
pos 50-85 XP 009009271.1 hypothetical protein HELRODRAFT 159110 Helobdella robusta
Annelid
Anopheles CKI
Squamate CDKN1A
Aves/Neodermata CDKN1A
pos 18-68 XP 067387698.1 cyclin-dependent kinase inhibitor 1 isoform X2 Emydura macquarii macquarii
Turtle CDKN1A
pos 18-68 XP 006125354.1 cyclin-dependent kinase inhibitor 1 Pelodiscus sinensis
Testudines
Crocodilian CDKN1A
Bony/Cartilaginous Fish CDKN1A
Mammalian CDKN1A
Amphibian CDKN1A
pos 21-71 XP 069095085.1 cyclin-dependent kinase inhibitor 1-like Pleurodeles waltl
Amphibia
pos 17-67 XP 064425187.1 cyclin-dependent kinase inhibitor 1 Latimeria chalumnae
Sarcopterygii
CDKN1D
2
142
3
2
28
10
3
7
11
7
2
165
261
4
3
2
11
60
8
2
4
21
4
3
186
69
13
234

Fig. S3.

Figure S3. Phylogenetic analysis of parasitic flatworm CKI suggests a horizontal transfer from a distantly related metazoan. Full consensus tree (1411 members) from maximum likelihood analysis of *S. mansoni* CKI. Left: CKI homologs generally fall into CDKN1A (blue), CDKN1B (red), CDKN1C (yellow), and CDKN1D groupings (dark blue). Homologs that did not cluster neatly within any of these groups are grouped together as “CKI” (green). The number next to each collapsed branch indicates how many members are in each branch. Right: Individual homologs are labeled with aligned residues, RefSeq accession, gene name, and species name. When possible, individual homologs were grouped together and labeled accordingly (e.g., “Bony Fish CDKN1B”). When clades did not group together into one or two clusters, they were instead labeled with colored shading (e.g., “Aves” in green shading). Invertebrate CKI molecules are labeled with red asterisks. The clade that contains avian CDKN1A along with parasitic flatworm CKI is indicated with a dashed red box. The black number next to each collapsed branch (triangle) indicates how many members there are in each branch. The number in the white box indicates the UFBoot bootstrap approximation value. Support values for branches with UFBoot support greater than 95 are not shown. Data are from 10 runs.

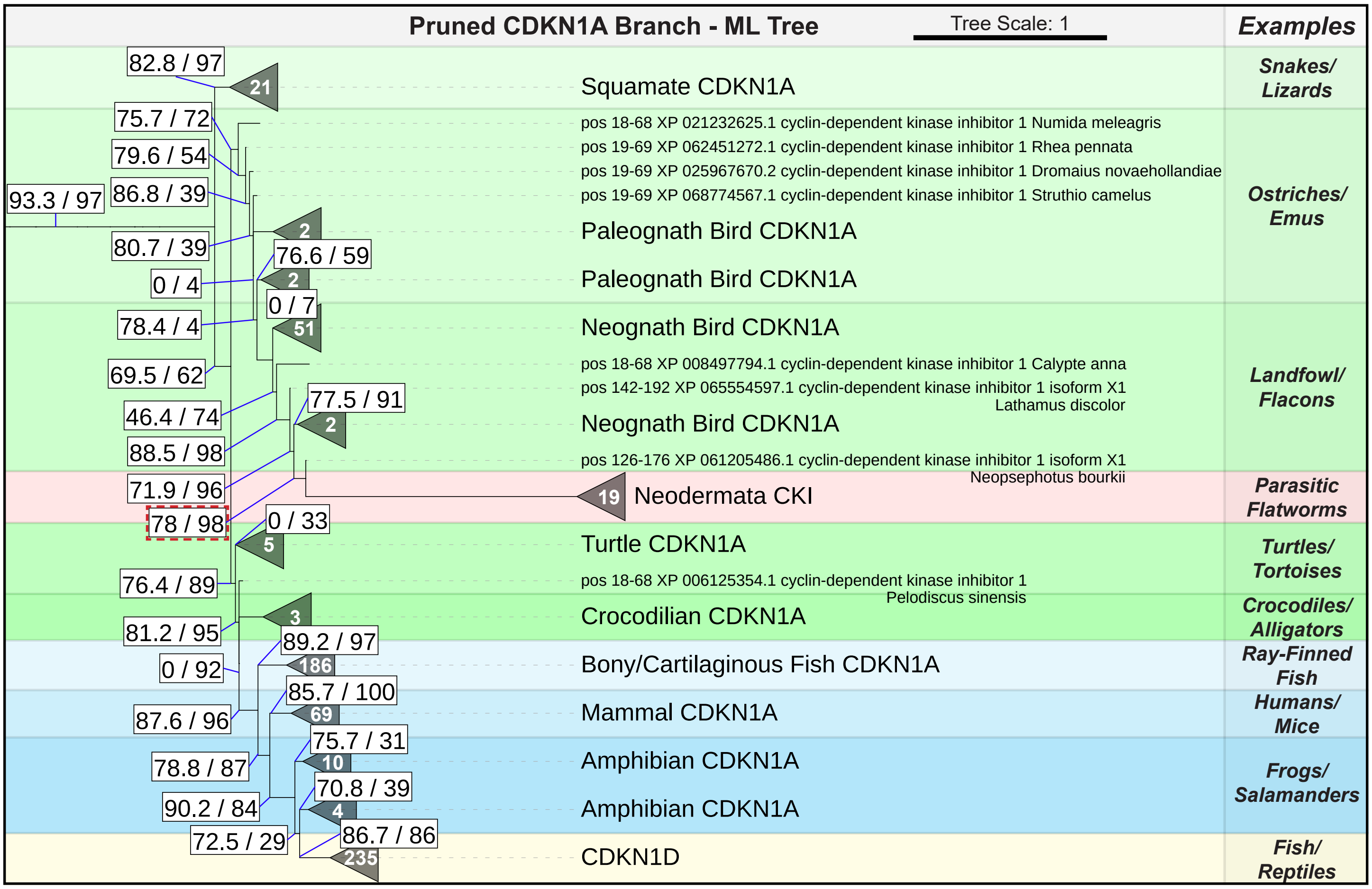

Pruned CDKN1A Branch - ML Tree
Tree Scale: 1
Examples
Squamate CDKN1A
Snakes/ Lizards
pos 18-68 XP 021232625.1 cyclin-dependent kinase inhibitor 1 Numida meleagris
pos 19-69 XP 062451272.1 cyclin-dependent kinase inhibitor 1 Rhea pennata
pos 19-69 XP 025967670.2 cyclin-dependent kinase inhibitor 1 Dromaius novaehollandiae
pos 19-69 XP 068774567.1 cyclin-dependent kinase inhibitor 1 Struthio camelus
Paleognath Bird CDKN1A
Paleognath Bird CDKN1A
Ostriches/ Emus
Neognath Bird CDKN1A
pos 18-68 XP 008497794.1 cyclin-dependent kinase inhibitor 1 Calypte anna
pos 142-192 XP 065554597.1 cyclin-dependent kinase inhibitor 1 isoform X1 Lathamus discolor
Neognath Bird CDKN1A
pos 126-176 XP 061205486.1 cyclin-dependent kinase inhibitor 1 isoform X1 Neopsephotus bourkii
Landfowl/ Flacons
Neodermata CKI
Parasitic Flatworms
Turtle CDKN1A
pos 18-68 XP 006125354.1 cyclin-dependent kinase inhibitor 1 Pelodiscus sinensis
Turtles/ Tortoises
Crocodilian CDKN1A
Crocodiles/ Alligators
Bony/Cartilaginous Fish CDKN1A
Ray-Finned Fish
Mammal CDKN1A
Humans/ Mice
Amphibian CDKN1A
Amphibian CDKN1A
Frogs/ Salamanders
CDKN1D
Fish/ Reptiles
82.8 / 97
75.7 / 72
79.6 / 54
93.3 / 97
86.8 / 39
80.7 / 39
76.6 / 59
0 / 4
0 / 7
78.4 / 4
69.5 / 62
77.5 / 91
46.4 / 74
88.5 / 98
71.9 / 96
78 / 98
0 / 33
76.4 / 89
81.2 / 95
89.2 / 97
0 / 92
85.7 / 100
87.6 / 96
75.7 / 31
78.8 / 87
70.8 / 39
90.2 / 84
72.5 / 29
86.7 / 86
21
2
2
51
2
19
5
3
186
69
10
4
235

Fig. S4.

Phylogeny of CKI within CDKN1A – Maximum Likelihood Tree. Pruned maximum likelihood phylogenetic tree (617 members) showing the clade of CDKN1A homologs from the phylogenetic analysis of *S. mansoni* CKI. The white number inside each collapsed branch (triangle) indicates how many members there are in each branch. The number in the white box indicates the Shimodaira–Hasegawa approximate Likelihood Ratio Test (SH-aLRT) value (left) and the UFBoot bootstrap approximation value (right). Support values for branches with SH-aLRT support greater than 95 are not shown. Data are from 10 runs.

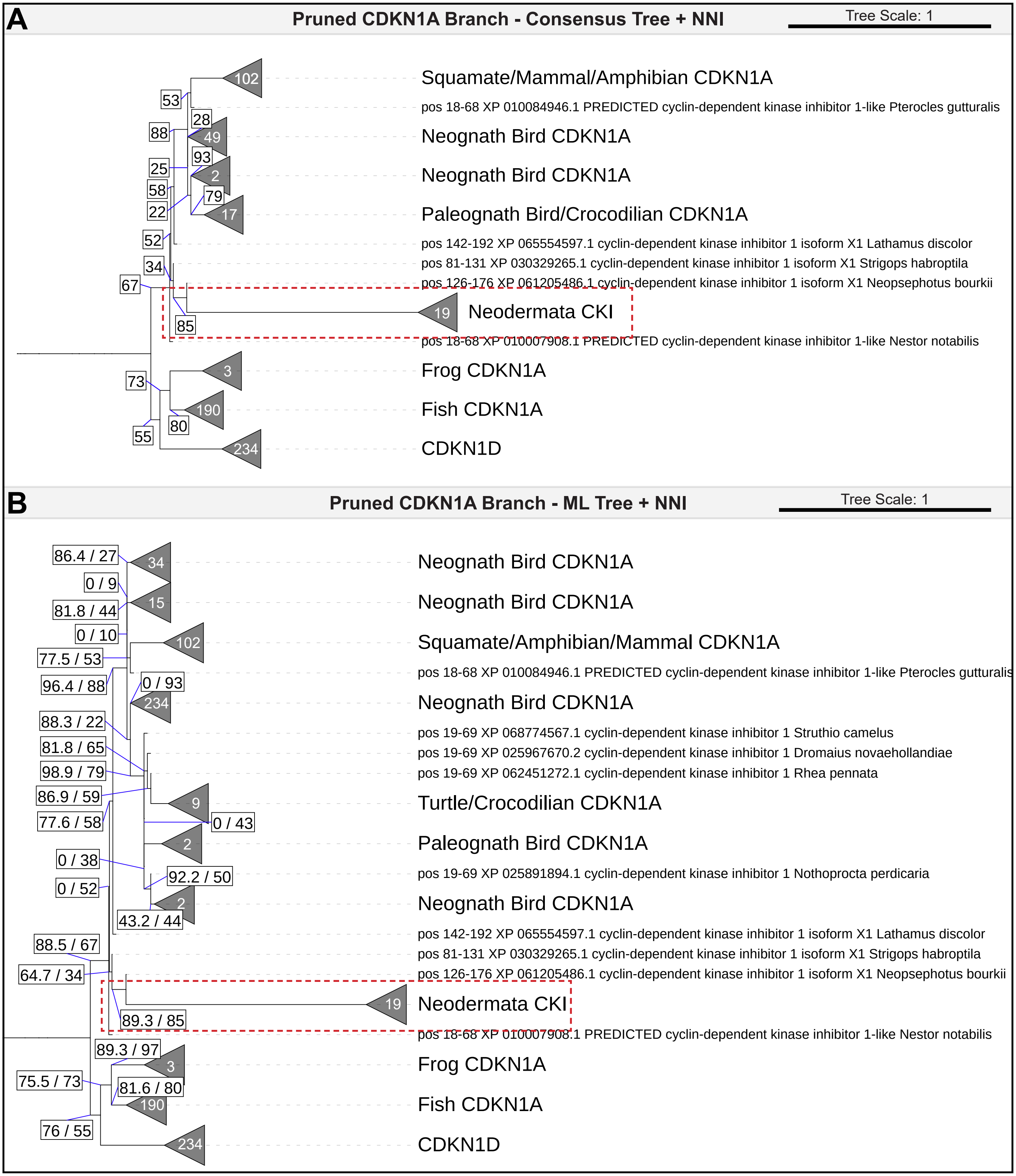
A
Pruned CDKN1A Branch - Consensus Tree + NNI
Tree Scale: 1
Squamate/Mammal/Amphibian CDKN1A
pos 18-68 XP 010084946.1 PREDICTED cyclin-dependent kinase inhibitor 1-like Pterocles gutturalis
Neognath Bird CDKN1A
Neognath Bird CDKN1A
Paleognath Bird/Crocodilian CDKN1A
pos 142-192 XP 065554597.1 cyclin-dependent kinase inhibitor 1 isoform X1 Lathamus discolor
pos 81-131 XP 030329265.1 cyclin-dependent kinase inhibitor 1 isoform X1 Strigops habroptila
pos 126-176 XP 061205486.1 cyclin-dependent kinase inhibitor 1 isoform X1 Neopsephotus bourkii
Neodermata CKI
pos 18-68 XP 010007908.1 PREDICTED cyclin-dependent kinase inhibitor 1-like Nestor notabilis
Frog CDKN1A
Fish CDKN1A
CDKN1D
B
Pruned CDKN1A Branch - ML Tree + NNI
Tree Scale: 1
Neognath Bird CDKN1A
Neognath Bird CDKN1A
Squamate/Amphibian/Mammal CDKN1A
pos 18-68 XP 010084946.1 PREDICTED cyclin-dependent kinase inhibitor 1-like Pterocles gutturalis
Neognath Bird CDKN1A
pos 19-69 XP 068774567.1 cyclin-dependent kinase inhibitor 1 Struthio camelus
pos 19-69 XP 025967670.2 cyclin-dependent kinase inhibitor 1 Dromaius novaehollandiae
pos 19-69 XP 062451272.1 cyclin-dependent kinase inhibitor 1 Rhea pennata
Turtle/Crocodilian CDKN1A
Paleognath Bird CDKN1A
pos 19-69 XP 025891894.1 cyclin-dependent kinase inhibitor 1 Nothoprocta perdicaria
Neognath Bird CDKN1A
pos 142-192 XP 065554597.1 cyclin-dependent kinase inhibitor 1 isoform X1 Lathamus discolor
pos 81-131 XP 030329265.1 cyclin-dependent kinase inhibitor 1 isoform X1 Strigops habroptila
pos 126-176 XP 061205486.1 cyclin-dependent kinase inhibitor 1 isoform X1 Neopsephotus bourkii
Neodermata CKI
pos 18-68 XP 010007908.1 PREDICTED cyclin-dependent kinase inhibitor 1-like Nestor notabilis
Frog CDKN1A
Fish CDKN1A
CDKN1D

Fig. S5.

Phylogeny of CKI within CDKN1A – With Nearest Neighbor Interchange. Pruned (621 members) (A) consensus or (B) maximum likelihood phylogenetic tree (generated using nearest neighbor interchange to correct for model violations) showing the clade of CDKN1A homologs from the phylogenetic analysis of *S. mansoni* CKI. The dashed red box indicates that branchpoint between avian CDKN1A and parasitic flatworm CKI. The white number inside each collapsed branch (triangle) indicates how many members there are in each branch. In (A), the number in the white box indicates the UFBoot bootstrap approximation value. Support values for branches with UFBoot support greater than 95 are not shown. In (B), the number in the white box indicates the Shimodaira–Hasegawa approximate Likelihood Ratio Test value (left) and the UFBoot bootstrap approximation value (right). Support values for branches with SH-aLRT support and UFBoot support greater than 95 are not shown. Data are from 10 runs.

Table S1. (separate file)

List of parasitic flatworm *CKI* homologs including the source organism, gene accession number(s), genomic location, amino acid sequence, upstream/downstream genes, and miscellaneous notes.

Table S2. (separate file)

List of flatworms available on NCBI that are at time of writing unannotated. Organism name, clade, and genome assembly are indicated. This table also contains the results of a tblastn search using S. mansoni, P. xenopodis, or H. microstoma CKI as a query against the indicated genome reported as the scaffold and e value of the hit.

Table S3. (separate file)

List of free-living flatworm TIMM21 homologs and the genes located downstream of these homologs.

Table S4. (separate file)

List of oligonucleotides used to produce riboprobes/dsRNA as well as primers used to perform qPCR.

Table S5. (separate file)

Accession numbers, organisms, and sequences used to generate the multiple sequence alignment in Fig S1A.

Table S6. (separate file)

Results of the DELTA-BLAST search performed as described in materials and methods. Iteration 3 (used for downstream analysis) is shaded green.

Table S7. (separate file)

FASTA format of the sequences of addtional CKI homologs from spiralians that did not appear in the initial DELTA-BLAST search.

Table S8. (separate file)

Output of the MAFFT-LAST algorithm used as described in the materials and methods.

Data S1. (separate file)

Newick format consensus tree of data shown in Fig. 4B and Fig. S3

Data S2. (separate file)
Newick format maximum likelihood tree of data shown in Fig. S4

Data S3. (separate file)
Newick format consensus tree of data shown in Fig. 5A

Data S4. (separate file)
Newick format maximum likelihood tree of data shown in Fig. 5B
